# Optimizing future Telehealth mental health programs: a secondary analysis of a prospective cohort study to identify key predictors of intervention response in the Telehealth intervention program for older adults (TIP-OA)

**DOI:** 10.1186/s12888-025-07688-1

**Published:** 2026-03-31

**Authors:** Christina Rigas, Paola Lavin, Chien-Lin Su, Mahdi Hassan, Karin Cinalioglu, Blanca Vacaflor, Elena Dikaios, Allana Goodman, Marim Ibrahim, Johanna Gruber, Jade Se, Neeti Sasi, Rim Nazar, Katie Bodenstein, Sasha Elbaz, Hannah Fajzel, Sonia Berkani, Cezara Hanganu, Helen Noble, Karl Looper, Stephane Bouchard, Dallas Seitz, Sanjeev Kumar, Olivier Beauchet, Cyrille Launay, Emily McDonald, Bassam Khoury, Andrew Ryder, Bruno Battistini, Pascal Fallavollita, Ipsit Vahia, Harmehr Sekhon, Syeda Bukhari, Soham Rej

**Affiliations:** 1 Department of Psychiatry, McGill University, Montreal, QC Canada; 2 Jewish General hospital/Lady Davis Institute, Montreal, QC Canada; 3 GeriPARTy Research Lab, Montreal, QC Canada; 4 School of Nursing and Midwifery, Queen’s University, Belfast, UK; 5 Department of Psychoeducation and Psychology, Université du Québec en Outaouais, Gatineau, QC Canada; 6 Department of Psychiatry, University of Calgary, Calgary, AB Canada; 7 Adult Neurodevelopmental and Geriatric Psychiatry Division, Centre for Addiction and Mental Health, Toronto, ON Canada; 8 McGill University Health Center Research Institute (RI-MUHC), Montreal, QC Canada; 9 Department of Educational and Counselling Psychology, McGill University, Montreal, QC Canada; 10 Interdisciplinary school of Health Sciences, Faculty of Health Sciences, University of Ottawa, Ottawa, ON Canada; 11 Interdisciplinary School of Health Sciences, University of Ottawa, Ottawa, ON Canada; 12 McLean Hospital, Harvard Medical School, Boston, MA USA

**Keywords:** Telehealth, Older adults, Volunteer-based intervention, Predictors of treatment response, Social isolation, Geriatric mental health

## Abstract

**Background:**

The Telehealth Intervention Program for Older Adults (TIP-OA) was a volunteer-based phone support program for to older adults during the COVID-19 pandemic. While volunteer-based phone programs can be effective in providing mental health support, there is limited data on the predictors of response to such interventions. This study aimed to examine clinical and demographic predictors of response to the TIP-OA intervention among older adults.

**Methods:**

This secondary analysis of a prospective cohort study included 82 TIP-OA users who expressed interest in the program’s research component, met inclusion criteria, and provided informed consent. Participants completed both baseline and 8-week assessments or had 4-week data carried forward using the last observation carried forward (LOCF) method. The intervention consisted of weekly supportive phone calls over eight weeks. Baseline mental health risk level was assessed by clinicians during intake and categorized as low, medium, or high based on symptom severity within specific symptom categories. Associations between baseline risk level and changes in stress (primary outcome), depression, and anxiety (secondary outcomes) were examined. The primary outcome was measured by the Perceived Stress Scale (PSS; scores ranging from 0 (never) to 4 (very often)). Secondary outcomes were measured by the Patient Health Questionnaire-9 (PHQ-9; scores ranging from 0 (not at all) to 3 (nearly every day)), and the Generalized Anxiety Disorder-7 scale (GAD-7; scores can range from 0 (not at all sure) to 3 (nearly every day)).

**Results:**

At 8 -week follow-up assessment, participants with higher baseline risk levels showed greater reductions in stress (mean difference in Perceived Stress Scale reduction by 2.13, (F(1,77) = 2.82, *p* = 0.09, 95% CI [−0.46, 5.56]) and depression (t(73) = −1.92, *p* = 0.059; Std. beta = −0.43, 95% CI [−0.87, 0.02]). Additionally, not identifying as a visible minority predicted lower stress scores, while male gender and a university education level were associated with greater reductions in depression scores.

**Conclusion:**

TIP-OA participants with higher baseline mental health risk appeared to benefit more in terms of reductions in stress and depression. Non-minority status, male gender, and university education were also associated with better post-intervention outcomes in depression. Future studies should explore predictors of response in similar intervention programs through larger confirmatory studies.

**Trial registration:**

Registered on clinicaltrials.gov (clinical trial no.: #NCT04523610) on 16/07/2020.

## Background

Approximately 40% of Canadian older adults faced social isolation during the COVID-19 pandemic [1]. This isolation has been linked to elevated levels of stress, depression, anxiety, and other mental health challenges [1, 2]. With growing concerns about the mental health effects of isolation in the older adult population and the limitations of in-person services, there has been a marked increase in the use of telehealth interventions to address their mental health needs [2, 3].

In response, our research team launched the Telehealth Intervention Program for Older Adults (TIP-OA) in March 2020. This program, offering free weekly phone calls from lay volunteers, targeted older adults (aged 60 and over) in Quebec, Canada. Since its inception, TIP-OA has enlisted over 300 volunteers who have provided support to more than 800 older adults throughout the province. Recently, we published the findings of an 8-week longitudinal study on the effects of TIP-OA on stress, depression, and anxiety during the pandemic, in TIP-OA users who were eligible for and consented to taking part in the research component of the program [2]. The study revealed that TIP-OA was linked to statistically significant reductions in depression and anxiety severity among participants who started with higher levels of these symptoms [2]. In contrast, the present study investigates predictors of change using clinician-rated mental-health risk levels—derived from structured intake assessments, alongside demographic characteristics. Throughout the pandemic, various telehealth interventions have been implemented to support older adults’ mental health, and several early studies have demonstrated their feasibility and effectiveness in improving psychological outcomes [4].

However, to date, no studies have examined the predictors of response to telehealth intervention programs for mental health in the geriatric population during the pandemic. Identifying such predictors would enable us to pinpoint the individuals most likely to benefit from these interventions and improve the design, implementation, and targeting of future programs [5, 6]. In this study, our primary goal was to determine whether participants’ baseline mental health risk levels were associated with greater reductions in stress (the primary outcome), as well as depression and anxiety (secondary outcomes). Additionally, we sought to identify demographic factors (age group, gender, marital status, living situation, education level, and self-identified visible minority status) that were linked to better primary and secondary mental health outcomes.

## Materials and methods

### Design, participants and procedures

#### Study design

This study is a secondary analysis of data collected in a previously conducted 8-week prospective cohort study of TIP-OA service users who were eligible and provided informed consent to participate in the research component of the program [2, 7]. This paper examines clinical and demographic variables to identify predictors of response to the intervention after 8 weeks. While our primary endpoint was 8 weeks, data were also collected at 4 weeks, and last-observation carried forward (LOCF) [8] was applied using 4-week data to address missing 8-week data. This study was registered on ClinicalTrials.gov (identifier: NCT04523610). While the current analysis represents a secondary observational investigation, the broader TIP-OA program was a prospective clinical study registered to ensure transparency of planned outcomes and analytic methods.

#### Participants

Individuals were referred or self-referred to the TIP-OA program through community organizations, primary care clinics, and public advertising during the COVID-19 pandemic. After initial contact, a clinician conducted an intake assessment to confirm eligibility and assign risk level before matching participants with volunteers. Participants who were identified as prospective participants for research (i.e. their file did not indicate any exclusion criteria) were contacted prior to receiving their first call from their assigned volunteer and invited to participate in the research component of the program. Participants met the following criteria: 1) TIP-OA users who provided informed consent regarding the research study 2) aged ≥ 60 years, 3) received ≤ 1 volunteer phone call prior to study enrollment, 4) living in Quebec, and 5) fluent in English or French. Exclusion criteria were: 1) acute (requiring immediate care) suicidal or psychotic thoughts (for which participants were referred to appropriate services), 2) severe hearing impairment, or 3) severe cognitive impairment.

#### Ethics

This study complied with the Declaration of Helsinki and was approved by the Jewish General Hospital Research Ethics Committee on September 24, 2020. The study was registered on clinicaltrials.gov (trial number: #NCT04523610) on July 16, 2020.

### Intervention: Telehealth intervention program for older adults (TIP-OA)

TIP-OA involved weekly friendly phone calls delivered by trained volunteers. Training consisted of a two-hour virtual active listening and support session conducted by clinicians, as well as a detailed training manual covering the material, sample conversations, and an extensive list of community resources. Volunteers promoted social interactions through active listening and helped connect participants to community social services. Participants received at least one phone call weekly for 8 weeks. For further details on TIP-OAs design and methodology, refer to the protocol paper [7].

### Outcomes and outcome measures

The primary outcome was stress, measured using the Perceived Stress Scale (PSS), a 14-item tool assessing the degree to which life events were perceived as stressful in the past month [9]. Secondary outcomes were depression and anxiety. Depression was assessed using the Patient Health Questionnaire-9 (PHQ-9), a 9-item self-report tool measuring depression severity based on the frequency of symptoms in the last two weeks [10]. Anxiety was measured using the Generalized Anxiety Disorder-7 scale (GAD-7), a 7-item questionnaire that evaluates anxiety symptoms over the past two weeks [11].

### Predictors of outcomes

#### Baseline mental health risk level

Baseline mental health risk level was assessed at intake by TIP-OA program clinicians, including licensed psychologists, social workers, and psychiatric nurses with experience in geriatric mental health. Assessments followed a standardized intake template and clinical interview guidelines developed for the program to ensure consistency of ratings across cases (details available in the Dikaios et al., 2020 paper). Clinicans considered multiple symptom categories reflecting psychological and functional vulnerability in older adults, including confusion, psychotic thoughts, depression or anxiety, suicidality, functional impairment, COVID-related distress, and other presenting concerns. Each symptom category (e.g., confusion, psychotic thoughts, depression or anxiety, suicidality, functional impairment, COVID-related distress) was first evaluated individually as none/mild, moderate, or severe using structured clinical judgment. These within-category evaluations were then considered in aggregate to assign an overall baseline risk level: low risk (predominantly none/mild across categories), medium risk (at least two categories judged moderate), and high risk (at least one category judged severe) [7]. For the purposes of the present secondary analysis, intake risk ratings were dichotomized into “low mental health risk level” (low risk) and “high mental health risk level” (medium or high risk) [7].

#### Demographic variables

Based on prior research [12, 13], we selected and analyzed demographic variables for potential associations with changes in mental health outcomes from baseline to 8 weeks. These variables included age group, gender, marital status, living situation, education level, and self-identified visible minority status. Detailed descriptions of these variables can be found in Table 1.

**Table 1:** Participant characteristics

Demographic Variables	Frequency (%) High Risk (n = 32)	Frequency (%) Low Risk (n = 50)	Frequency (%) All Participants (n = 82)
**Baseline Mental Health Risk Level (n = 82)**			
Low Risk	-	-	50 (61.0)
High Risk	-	-	32 (39.0)
**Age Group (n = 82)**			
60–69 (*n* = 22)	9 (28.1)	13 (26.0)	22 (26.8)
70–79 (*n* = 37)	14 (43.8)	23 (46.0)	37 (45.1)
80+ (*n* = 23)	9 (28.1)	14 (28.0)	23 (28.0)
**Gender (n = 82)**			
Male (*n* = 24)	6 (18.8)	18 (36.0)	24 (29.3)
Female (*n* = 58)	26 (81.2)	32 (64.0)	58 (70.7)
**Education Level (n = 82)**			
Elementary graduate or unsure (*n* = 14)	7 (21.9)	7 (14.0)	14 (17.1)
Highschool (*n* = 35)	16 (50.0)	19 (38.0)	35 (42.7)
University (*n* = 33)	9 (28.1)	24 (48.0)	33 (40.2)
**Marital Status (n = 82)**			
Single (*n* = 36)	15 (46.8)	21 (42.0)	36 (44)
Married or common-law (*n* = 23)	6 (18.8)	17 (34.0)	23 (28.0)
Separated (*n* = 23)	11 (34.4)	12 (24.0)	23 (28.0)
**Living Situation (n = 82)**			
Alone (*n* = 63)	27 (84.4)	36 (72.0)	63 (76.8)
Support (*n* = 19)	5 (15.6)	14 (28.0)	19 (23.2)
**Self-Identifying as Visible Minority (n = 82)**			
No (*n* = 72)	30 (93.8)	42 (84.0)	72 (87.8)
Yes (*n* = 10)	2 (6.2)	8 (16.0)	10 (12.2)

### Statistical analyses

Baseline demographic variables (n, frequency, %) and baseline mental health risk levels were detailed to characterize the study sample (Table 1). One-way Analysis of Variance (ANOVA) [14] was conducted to assess statistically significant differences between mean changes in stress (primary outcome), depression, and anxiety (secondary outcomes) scores across risk levels and demographic variable groups. Normality of outcome scores was assessed using the Kolmogorov-Smirnov normality test [15]. All ANOVAs were two-tailed, with alpha = 0.05 [14], and effect sizes (η2) with 95% confidence intervals (CIs) were calculated based on one-way ANOVA [14]. Effect sizes were categorized as small (0.01), medium (0.06), and large (0.14) [16].

Multiple linear regression analyses were then conducted with variables that were either significant or approached significance to assess whether baseline mental health risk level independently predicted changes in primary and secondary outcomes [17]. Statistical significance was set at two-tailed alpha = 0.05 (17). LOCF was used to address missing 8-week data due to attrition or incomplete questionnaires [8] Statistical analyses were performed using SPSS (version 28.0; SPSS, Inc., Chicago, IL) [18] and R (Version 3.5, R Foundation for Statistical Computing, Vienna, Austria) [19].

#### Results

### Study sample

**Fig. 1:**
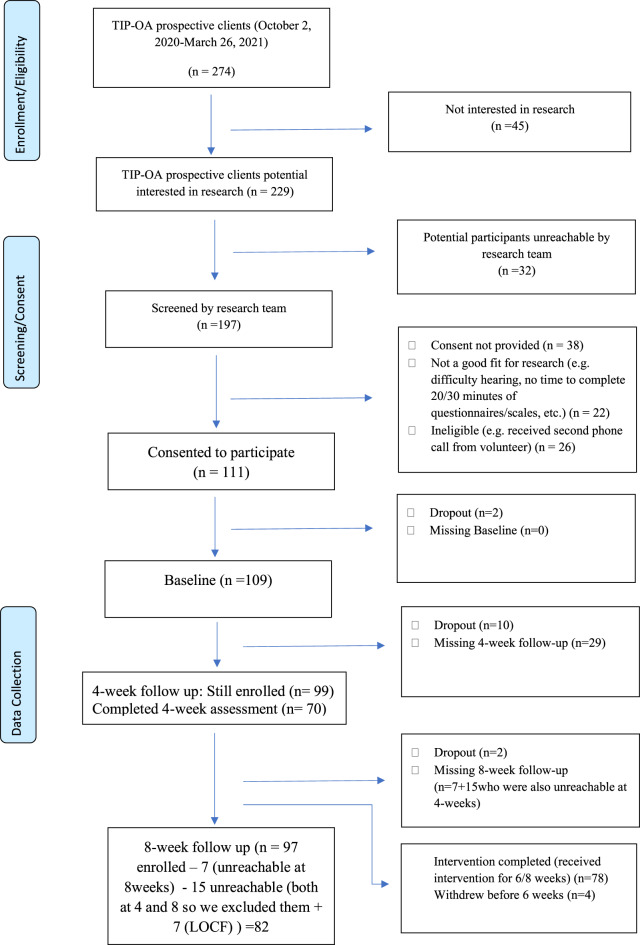
Consort diagram

As shown in Fig. 1, a total of 274 potential participants were identified, of whom 229 were contacted for research participation. Thirty-two were unreachable, and 197 were screened. Of those screened, 38 did not provide consent, 22 were not a good fit (e.g., difficulty hearing, no time to complete questionnaires), and 26 were ineligible, resulting in 111 participants who consented to participate (48.47%). Two participants dropped out prior to baseline, leaving 109 who provided baseline data. Of these, 78 completed ≥75% of the intervention sessions (a minimum of 6 out of 8 weeks), with an attrition rate of 12% (*n* = 14/111, 12.61%). At the 4-week point, 70 participants completed data collection, and at 8 weeks, 75 completed follow-up. Seven participants who completed the 4-week but not the 8-week assessment were included using the last-observation-carried-forward (LOCF) method, yielding a total analytic sample of 82 (75 with complete 8-week data and 7 imputed cases). Analyses were conducted on participants with at least one post-baseline assessment, with imputation applied only for those missing 8-week data. Analyses examined changes from baseline to 8 weeks (post-intervention) only. Baseline mental health risk levels and demographic characteristics of the sample are provided in Table 1.

**Fig. 2:**
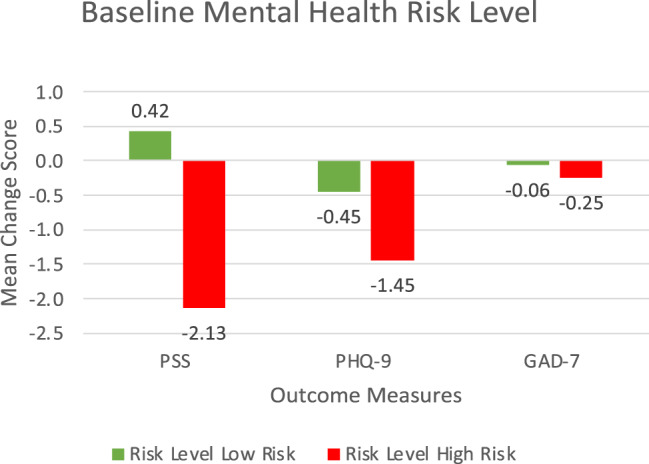
Baseline mental health risk change scores at 8-weeks. abbreviations: PSS: perceived stress scales, PHQ-9: patient health questionnaire-9, GAD-7:Generalized anxiety disorder-7

For the following results, mean changes in outcome scores are presented in Table 2 and Fig. 2. One-way ANOVA results are presented in Table 3. Multiple linear regression results are presented in Table 4.

**Table 2:** Mean changes in outcome scores

Variable	PSS Mean change scores (St. Dev.)	PHQ-9 Mean change scores (St. Dev.)	GAD-7 Mean change scores (St. Dev.)
**Risk Level**			
Low Risk	0.42 (1.02)	−0.45 (0.69)	−0.06 (0.84)
High Risk	−2.13 (1.02)	−1.45 (0.81)	−0.25 (0.52)
**Age Group**			
Age 60–69	−2.48 (1.25)	−1.41 (0.76)	−0.05 (0.66)
Age 70–79	0.57 (0.97)	−0.73 (0.74)	0.29 (1.00)
Age 80+	0.06 (1.99)	−0.11 (1.32)	−0.22 (0.99)
**Gender**			
Male	−0.37 (1.77)	−2.57 (1.13)	−1.50 (1.23)
Female	−0.67 (0.76)	−0.13 (0.51)	0.39 (0.57)
**Visible Minority Status**			
No	−1.28 (0.76)	−0.07 (0.55)	−0.17 (0.60)
Yes	4.20 (2.24)	−0.44 (1.22)	0.22 (1.28)
**Living situation**			
Alone	−0.63 (0.85)	−0.95 (0.55)	0.03 (0.60)
With Support	−0.42 (1.59)	−0.44 (1.22)	−0.68 (1.21)
**Marital Status**			
Single	−1.71 (1.10)	−1.59 (0.69)	−0.44 (0.79)
Married/Common-law	0.05 (1.49)	−1.24 (1.10)	−0.62 (1.12)
Separated	0.59 (1.38)	0.61 (0.90)	0.82 (1.00)
**Education Level**			
Elementary	−1.91 (1.41)	0.93 (1.35)	0.33 (0.62)
High school	0.58 (0.99)	−0.41 (0.68)	−0.26 (0.75)
University	−1.58 (1.38)	−2.17 (0.82)	−0.19 (1.08)

Abbreviations: PSS: Perceived Stress Scales, PHQ-9: Patient Health Questionnaire-9, GAD-7: Generalized Anxiety Disorder-7

**Table 3:** One-way ANOVAs

Variable			PSS		PHQ-9				GAD-7		
		Test Statistic	P- value	_**η**_**2**	Test Statistic		p-value	_**η**_**2**	Test Statistic	P-value	_**η**_**2**
**Age Group**		F(2,76) = 1.26	0.28	0.03	F(2,75) = 1.22		0.30	0.03	F(2,76) = 0.99	0.37	0.03
**Gender**		F(1,77) = 0.03	0.85	0.00	F(1,76) = 5.03		0.02	0.06	F(1,77) = 2.47	0.12	0.03
**Visible Minority Status**		F(1,77) = 6.31	0.01	0.08	F(1,76) = 1.37		0.24	0.02	F(1,77) = 0.05	0.81	0.00
**Education Level**		F(2,74) = 1.06	0.35	0.03	F(2,75) = 2.67		0.07	0.07	F(2,75) = 0.06	0.93	0.00
**Marital Status**	F(2,76) = 0.94		0.39	0.02		F(2,75) = 1.79	0.17	0.05	F(2,76) = 0.60	0.54	0.02
**Living situation**	F(1,77) = 0.01		0.90	0.00		F(1,76) = 0.18	0.66	0.00	F(1,77) = 0.31	0.57	0.00

Abbreviations: PSS: Perceived Stress Scales, PHQ-9: Patient Health Questionnaire-9, GAD-7: Generalized Anxiety Disorder-7

**Table 4:** Multiple linear regression

Independent Variable	Estimate	Std. Error	t-value	p-value	95% Confidence Interval
**PSS (n = 72)**					
Intercept	4.60	2.04	2.25	0.02	0.52, 8.68
High Risk Level	−2.03	1.49	−1.36	0.17	−5.00, 0.94
Not Visible Minority	−5.02	2.19	−2.29	0.02	−9.39, −0.66
**PHQ-9 (n = 78)**					
Intercept	2.50	1.26	1.97	0.05	−0.01, 5.02
High Risk	−1.92	1.00	−1.92	0.05	−3.91, 0.07
Male Gender	−2.85	1.06	−2.67	0.00	−4.98, −0.72
High School	−1.05	1.33	−0.79	0.43	−3.72, 1.61
University	−3.33	1.37	−2.43	0.01	−6.06, 0.59
**GAD-7 (n = 79)**					
Intercept	0.50	0.78	0.63	0.52	−1.06, 2.07
High Risk Level	−2.27	1.10	−0.25	0.80	−2.47, 1.91
Male Gender	−1.90	1.20	−1.57	0.11	−4.30, 0.50

Abbreviations: PSS: Perceived Stress Scales, PHQ-9: Patient Health Questionnaire-9, GAD-7: Generalized Anxiety Disorder-7

### Stress (perceived stress Scale; PSS)

The one-way ANOVAs revealed no statistically significant difference in mean PSS change scores between the low and high-risk groups at 8 weeks (F(1,77) = 2.82, *p* = 0.09, 95% CI [−0.46, 5.56]), with a small effect size (η2 = 0.04). The low-risk group had a mean change of +0.42 (1.02), while the high-risk group had a mean change of −2.13 (1.02). However, there was a significant difference in PSS change scores between visible minority status groups (F(1,77) = 6.31, *p* = 0.01, 95% CI [0.008, 1.00]), with an effect size of η2 = 0.08. Non-visible minority participants had a mean change of −1.28 (0.76), while visible minority participants had a mean change of +4.20 (2.24).

Baseline risk level and visible minority status were included in the subsequent multiple linear regression model. The model testing whether these factors predicted PSS change at 8 weeks was significant (F(2, 76) = 4.12, *p* = 0.020, adj. R2 = 0.07). In this model, high-risk level did not significantly predict PSS changes at 8 weeks (beta = −2.03, 95% CI [−5.00, 0.94], t(76) = −1.36, *p* = 0.177; Std. beta = −0.31, 95% CI [−0.75, 0.14]). However, not identifying as a visible minority significantly predicted reduced PSS scores (beta = −5.03, 95% CI [−9.39, −0.66], t(76) = −2.29, *p* = 0.025; Std. beta = −0.76, 95% CI [−1.41, −0.10]).

### Depression (patient health questionnaire-9; PHQ-9)

The one-way ANOVAs indicated no statistically significant difference in mean PHQ-9 change scores between risk level groups at 8 weeks (F(1,76) = 0.93, *p* = 0.33, 95% CI [−1.06,3.06], η2 = 0.01). The low-risk group had a mean change of −0.45 (0.64), while the high-risk group had a mean change of −1.45 (0.81). However, there were significant differences in PHQ-9 change scores between gender groups (male vs. female), (F(1,76) = 5.03, *p* = 0.02, 95% CI [0.003,1.00]), with a medium effect size (η2 = 0.06). Males had a mean change of −2.57 (1.13) on the PHQ-9, while females had a mean change of −0.13 (0.51). The differences between education levels (elementary, high school, university) approached significance (F(2,75) = 2.67, *p* = 0.07, 95% CI [0.00,1.00]), with a medium effect size (η2 = 0.07). Participants with elementary-level education had a mean change of +0.93 (1.35), high school participants had a mean change of −0.41 (0.68), and university-educated participants had a mean change of −2.17 (0.82).

Subsequently, baseline risk level, gender, and education level were included in a multiple linear regression model. The regression model assessing if baseline risk level, gender, and education level significantly predicted PHQ-9 score changes at 8 weeks was significant (F(4,73) = 3.78, *p* = 0.008, adj. R2 = 0.13). While high-risk level did not significantly predict PHQ-9 changes, the p-value was close to significance (beta = −1.92, 95% CI [−3.91, 0.07], t(73) = −1.92, *p* = 0.059; Std. beta = −0.43, 95% CI [−0.87, 0.02]). Male gender significantly predicted greater reductions in PHQ-9 scores (beta = −2.86, 95% CI [−4.98, −0.73], t(73) = −2.68, *p* = 0.009; Std. beta = −0.64, 95% CI [−1.11, −0.16]). University education level also significantly predicted PHQ-9 reductions (beta = −3.33, 95% CI [−6.06, −0.60], t(73) = −2.43, *p* = 0.018; Std. beta = −0.74, 95% CI [−1.35, −0.13]).

### Anxiety (generalized anxiety disorder-7; GAD-7)

The one-way ANOVAs indicated no statistically significant difference in mean GAD-7 change scores at 8 weeks between the risk level groups (F(1,7) = 0.02, *p* = 0.86, 95% CI [−2.02, 2.40], η2 = 0.000). The low-risk group had a mean change of −0.06 (0.84) on the GAD-7, while the high-risk group had a mean change of −0.25 (0.52). Additionally, no statistically significant differences in GAD-7 change scores were found between any of the demographic groups.

The difference in GAD-7 scores between gender groups was closest to significance (F(1,77) = 2.47, *p* = 0.12, 95% CI [0.00, 1.00]), and thus it was included in the subsequent regression model along with baseline risk level. Males had a mean change of −1.5 (1.23), while females had a mean change of +0.39 (0.57). However, the regression model testing whether baseline risk level and gender predicted GAD-7 score changes at 8 weeks was not significant (F(2, 76) = 1.26, *p* = 0.291, adj. R2 = 0.006). Neither risk level nor gender significantly predicted changes in GAD-7 scores at 8 weeks.

## Discussion

To our knowledge, this is the first study to examine predictors of response to a volunteer-delivered telehealth intervention for the mental health of older adults during the pandemic. This study identified clinical and demographic factors associated with changes in stress, depression, and anxiety after 8 weeks of the TIP-OA intervention. Participants in the high mental health risk group tended to experience greater reductions in stress and depression over the 8-week period. Additionally, non-visible minority participants, males, and those with university education showed greater improvements in specific mental health outcomes post-intervention.

Our findings suggest that TIP-OA was particularly beneficial for older adults with higher baseline mental health risk, though further research with larger sample sizes is necessary to confirm this. Limited literature exists on volunteer-based and/or telehealth mental health interventions for older adults [4, 20], but our results align with studies showing that psychosocial or psychotherapeutic interventions tend to be more effective for individuals with higher baseline mental health risk [21–24]. Even though TIP-OA did not involve professional therapists or psychotherapeutic elements, it provided significant mental health support, particularly for those with higher risk. These findings emphasize that high baseline risk should not preclude older adults from participating in similar mental health intervention programs.

Our study also found that not identifying as a visible minority predicted greater reductions in stress scores. This may reflect existing disparities in mental health outcomes for racial and ethnic minorities in North America, especially during COVID-19, where these groups experienced higher rates of infection, hospitalization, and death [25]. Consistent with Statistics Canada data from 2020, visible minorities reported poorer mental health than non-immigrant European Canadians/White Americans [25]. Although TIP-OA did not have adverse effects on visible minority participants, our findings suggest that its current format may be more effective for non-minorities, at least regarding stress reduction. Racial and ethnic minorities often face additional stressors such as discrimination, socioeconomic disadvantage, and limited access to culturally sensitive care, all of which can contribute to poorer mental health outcomes [26]. These factors may also influence engagement with telehealth-based support programs. This highlights the need for interventions that incorporate culturally tailored approaches and address the specific needs of diverse communities.

Furthermore, our results showed that individuals with a university-level education experienced greater reductions in depression scores following the intervention. This aligns with extensive cross-national evidence demonstrating that higher educational attainment is consistently linked to better mental health and overall well-being in later life. Studies across diverse populations show that education contributes to improved emotional resilience, lower depression risk, and stronger self-rated health [27, 28]. These associations are thought to reflect the role of education in enhancing cognitive and social resources, health literacy, and engagement with preventive and therapeutic interventions. Accordingly, individuals with higher education may be better equipped to engage with and benefit from programs like TIP-OA, underscoring the importance of designing interventions that are inclusive and accessible to participants across all educational backgrounds.

This finding is consistent with epidemiological data showing that older adult females experience higher prevalence and greater chronicity of depression than males [29]. While gender differences in response to depression-interventions are mixed, possible mechanisms include gender-specific help-seeking behaviours, symptom-expression patterns (women more likely to report internalizing symptoms such as depression and anxiety, men more likely to benefit from structured social-contact and routine-based engagement), and different intervention-engagement styles [30, 31]. Our results suggest that structured telehealth support may have particular relevance for older men, and highlight the importance of exploring gender-related mechanisms of intervention response and designing delivery modes that meet the needs of both men and women equally.

## Strengths & limitations

This study had several strengths. Our sample was heterogeneous and representative of Montreal’s multicultural population, with participants recruited from community-based clinics, hospitals, and self-referrals. The study included a wide range of participants in terms of psychiatric/physical diagnoses, ethnicity, gender, and education, enhancing the generalizability of our findings [7]. However, the relatively small sample size and limited participation rate (48% of contacted individuals consented) may have introduced bias, as those who agreed to participate could differ in important ways from those who declined. This potential selection bias, together with the absence of longer-term follow-up data, limits the ability to determine whether the observed effects would persist over time or generalize to broader populations. Another limitation was that only English- and/or French-speaking participants were included, though the inclusion of French-speaking participants (Quebec’s official language) enhances the relevance of our results. Finally, as data were collected during the COVID-19 pandemic, participants’ stressors and social contexts may differ from those in non-pandemic times, which may limit generalizability to future or non-crisis settings. Future research should consider randomized controlled trials (RCTs) with larger and more diverse samples, longer follow-up periods, and control arms to further evaluate the effectiveness of telehealth interventions for older adults’ mental health. Future research should consider randomized controlled trials (RCTs) with larger and more diverse samples, longer follow-up periods, and control arms to further evaluate the effectiveness of telehealth interventions for older adults’ mental health.

## Conclusions

This study examined clinical and demographic predictors of response in stress, depression, and anxiety following participation in the TIP-OA program. Older adults with higher baseline mental health risk showed greater reductions in stress and depression over 8 weeks. Additionally, participants who were not visible minorities, males, and those with a university education showed greater improvements in specific mental health outcomes. These findings highlight potential predictors of response to a volunteer-based telehealth intervention and underscore the need for further research to understand the mechanisms underlying these associations. Future adaptations of the program could focus on improving inclusivity and engagement among underrepresented or disadvantaged participants, such as visible minorities and those with lower education levels, through culturally sensitive training, multilingual materials, and tailored support strategies. Matching volunteers and participants by language or cultural background, when possible, may further enhance comfort and engagement, ensuring equitable access to mental health support through telehealth programs like TIP-OA. Overall, these results are exploratory and should be interpreted with caution. Future randomized controlled trials are warranted to confirm these associations and further explore predictors of response in similar programs.

## Data Availability

Data is provided within the manuscript. Access to the data used can be made available upon request.
